# Review of Selected 2-Phenylethylamine Derivatives and Opioids, Systematic Review of Their Effects on Psychomotor Abilities and Driving Performance: Psychopharmacology in the Context of Road Safety

**DOI:** 10.3390/ph18101555

**Published:** 2025-10-16

**Authors:** Kacper Żełabowski, Kamil Biedka, Wojciech Pichowicz, Maria Sterkowicz, Izabela Radzka, Ignacy Ilski, Michał Wesołowski, Kacper Wojtysiak, Wiktor Petrov, Dawid Ślebioda, Maciej Rząca, Agnieszka Chłopaś-Konowałek

**Affiliations:** 1 Scientific Society for Psychopharmacology, Department of Forensic Medicine, Wroclaw Medical University, 4 J. Mikulicza-Radeckiego Street, 50-345 Wroclaw, Poland; kacper.zelabowski@outlook.com (K.Ż.); wojciech.pichowicz@outlook.com (W.P.); maria.sterkowicz@student.umw.edu.pl (M.S.); izabela.radzka@student.umw.edu.pl (I.R.); ignacy.ilski@student.umw.edu.pl (I.I.); kacper.wojtysiak@student.umw.edu.pl (K.W.); wiktor.petrov@student.umw.edu.pl (W.P.); dawid.slebioda@student.umw.edu.pl (D.Ś.); maciej.rzaca@student.umw.edu.pl (M.R.); 2 Department of Physiology and Pathophysiology, Division of Pathophysiology, Wroclaw Medical University, Chalubinskiego 10, 50-368 Wroclaw, Poland; kamil.biedka@umw.edu.pl (K.B.); michal.wesolowski@umw.edu.pl (M.W.); 3 Department of Forensic Medicine, Division of Molecular Techniques, Wroclaw Medical University, Sklodowskiej-Curie 52, 50-369 Wroclaw, Poland

**Keywords:** psychopharmacotherapy, drug abuse, psychoactive substances, psychomotor performance, driving safety, 2-Phenylethylamine derivatives, methylphenidate, ADHD, SUD

## Abstract

**Background:** Driving is a coordinated psychomotor activity that involves reaction time, attention, and decision-making. Psychoactive substances such as 2-phenylethylamine (PEA) derivatives and opioids may affect these functions and contribute to traffic safety. This systematic review revealed the effects of the selected PEA derivatives and opioids on psychomotor performance among drivers and potential road safety outcomes. **Methods:** The review followed PRISMA 2020 standards. Using the PICO method, we conducted a systematic search in Embase, PubMed, and Web of Science (2000–2025). Included studies involved adult participants and quantified the effect of PEA derivatives or opioids on driving-related psychomotor function. Thirty-one articles, such as randomized controlled trials, crossover studies, observational studies, and simulator-based studies, were examined. Risk of bias was evaluated using the RoB2 tool. **Results:** Evidence indicates therapeutic amphetamine and methylphenidate doses can enhance psychomotor function and safety in patients with ADHD. Recreational or high-dose use of methamphetamine and MDMA is associated with impaired coordination, variable speed, and increased impulsivity. Opioid effects are tolerance- and dose-dependent. Small therapeutic doses of fentanyl in chronically treated patients do not notably impair driving. On the other hand, methadone and tramadol commonly cause somnolence, retardation of reaction, and increased accident risk. **Conclusions:** The impact of opioids and PEA derivatives on psychomotor function is multifactorial, depending on dose, time, route of administration, and patient status. These substances can either improve or impair driving safety. The findings confirm the need for individual-specific pharmacotherapy treatment. They also highlight the importance of further studies to formulate evidence-based clinical and legislative guidelines.

## 1. Introduction

Driving is a complex behavior that requires constant processing of perceived stimuli both inside the individual and in the environment. Safe driving also depends on coordination between numerous cognitive skills, including planning, flexibility in thinking, and processing speed. The use of some drugs, including psychoactive chemicals, can reduce cognitive skills. This deterioration might reduce safe driving psychomotor skills, including decision-making, perception of distance, and staying in the lane [1]. Among such substances is 2-phenylethylamine (PEA), which is an endogenous neuroactive trace amine found in the human central nervous system. It acts as a neuromodulator, producing an enhancing and rewarding effect, which it owes to its ability to activate dopaminergic neurotransmission [2]. PEA is an integral component of both basic stimulants and psychoactive drugs. This study focuses on phenethylamine derivatives, specifically those that function as stimulants and hallucinogens [3]. We will exclude derivatives from the cathinones group; after reviewing the literature, none of the studies on cathinones met the criteria for inclusion in our review. Opioids, which are not PEA derivatives, will be analyzed separately for their impact on driving performance. Administration of phenylethylamine derivatives in safe therapeutic doses has been reported to enhance concentration and cognitive abilities. A representative example is the use of methylphenidate in the treatment of Attention-Deficit/Hyperactivity Disorder (ADHD). Individuals with ADHD are characterized by inattention, impulsivity, and hyperactivity [4]. The coexistence of ADHD with comorbid conditions poses a significant challenge in clinical management. Patients diagnosed with ADHD are more susceptible to developing an addiction, particularly to stimulant medications such as methylphenidate and amphetamine [4].

In recent decades, there has been an increasing interest in psychotropic substances, both in terms of their medical and recreational use. Between 2010 and 2021, the percentage of overdose deaths caused by fentanyl and stimulants (e.g., cocaine, methamphetamine) in the United States increased from 0.6% to 32.3% [5]. The most significant increase in overdose mortality, referred to as the fourth-wave phenomenon, was observed after 2015 [6]. The surge of interest in psychoactive substances observed since 2015 has prompted a re-examination impact of opioids and 2-phenylethylamine derivatives on psychomotor abilities in the context of road safety among drivers. In the United States, the period between 1999 and 2018 saw a total of 750,000 fatalities resulting from drug overdoses. The majority of these fatalities occurred subsequent to the ingestion of opioids. Furthermore, a 6.5% increase in opioid overdoses was observed between December 2018 and December 2019, marking an escalation from 47,096 cases to 50,177 cases [7].

Driving under the influence of drugs (DUID) has emerged as a significant concern. According to a 2022 study, DUID is most common in the age group of 18 to 25 years old—34% of study participants, and 64% of them were males [8]. Among the identified compounds are opioids and numerous phenylethylamine derivatives, such as MDMA and amphetamines, which have been shown to differentially affect psychomotor function, coordination, and decision-making. A comprehensive examination of the effects of these substances on driving is imperative for the development of effective regulatory frameworks and the formulation of safe medical guidelines for patients. It is also essential in efforts to counter risky driving behavior and improve road safety.

The objective of this study is to examine and understand the impact of specific 2-phenylethylamine derivatives and opioids on psychomotor abilities and their ramifications for traffic safety. Both patients receiving prescribed doses of 2-phenylethylamine derivatives and individuals using them recreationally often drive cars on a daily basis. This may pose a significant risk to road safety. However, the effects of these substances at therapeutic doses differ from those observed with recreational use, prompting an in-depth evaluation of the issue. The analysis will encompass the neuropharmacology, effects on the nervous system, and potential adverse effects associated with their use. A comprehensive review of epidemiological studies on the effects of opioids and 2-phenylethylamine derivatives on driving is presented.

### 1.1. Characterization of 2-Phenylethylamine, Its Derivatives and Opioids

**Figure 1:**
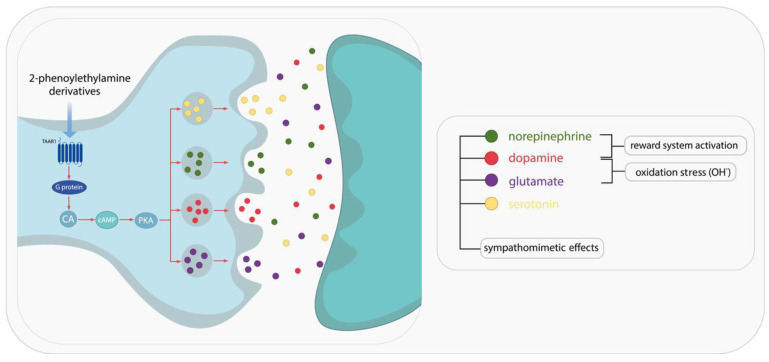
The effect of 2-phenylethylamine derivatives and TAAR1 agonists on monoamine transporters and neurotransmission.

2-Phenylethylamine (PEA, also known as β-phenylethylamine) is a naturally occurring amine that has been detected in trace amounts in the brains of humans and other mammals [9]. The presence of the substance has also been confirmed in cocoa products, cheese, and wine [10]. PEA (C_8_H_11_N) consists of a benzene ring linked to a two-carbon alkyl chain ending in an amino group. PEA is a fundamental component of basic stimulants and psychoactive drugs [3]. 2-Phenylethylamine is distinguished from amphetamine by the absence of a methyl group. This difference is related to the similar effects of the two compounds on their respective G protein receptors called TAARs. This similarity has sparked interest in this compound and its derivatives [9,11]. The precursor of L-phenylethylamine (PEA) is L-phenylalanine, which undergoes a process of enzymatic decarboxylation. This process is catalyzed by L-aromatic amino acid decarboxylase (L-AAD). PEA is rapidly metabolized by monoamine oxygenase B (MAO-B), which breaks it down to phenylacetic acid, a compound with effects similar to endogenous endorphins. The elimination half-life of PEA is approximately five to ten minutes [9,12,13]. The effectiveness of PEA in improving mood in depression cases treated with a selective MAO-B inhibitor is attributable to its high sensitivity to monoamine oxidase B [12]. PEA is stored in the synaptic vesicles of dopaminergic neurons and is released in response to depolarization and stimulation of the nigrostriatal dopaminergic pathway. PEA’s hydrophobic nature enables it to cross the blood–brain barrier easily, leading to its accumulation within neurons against the concentration gradient [10,11]. Trace amines, such as 2-phenylethylamine, are potent neuromodulators that affect the activity of other neurotransmitters. PEA and its derivatives act by directly stimulating monoamine transporters, including DAT, SERT, NET, and VMAT2. These transporters regulate the activity of neurotransmitters. The stimulation of these transporters has been demonstrated to induce sympathomimetic effects. PEA and its derivatives also elicit effects indirectly through their action as trace amine-associated receptor 1 (TAAR1) agonists [14]. The mechanism of action of these compounds involves altering the function of the monoamine transporters, thereby inhibiting serotonin, dopamine, epinephrine, and norepinephrine. An example of a potent TAAR1 agonist is methamphetamine (METH); it elicits a significant increase in cAMP concentration, thereby activating the adenylyl cyclase signaling cascade (Figure 1). This, in turn, has a marked effect on dopamine and glutamate transporters present within dopaminergic neurons [11]. The effect is analogous to that of direct inhibitors of dopamine transporters, such as methylphenidate [15,16].

It has been associated with sympathomimetic, “amphetamine-like” effects on appetite, focus, and mood. Activation of TAAR1 enhances dopamine sensitivity. Conversely, a mutation leading to TAAR1 deletion augments sensitivity to psychotropic substances and has been associated with schizophrenia [9,12,13]. TAAR1 has been demonstrated to play an important role in modulating the reward system and limbic system. This finding provides a rationale for exploring the potential of TAAR1 as a therapeutic target for the treatment of schizophrenia, depression, and drug addiction [10,15]. Amphetamine-like effects are also associated with an increase in oxidative stress, caused by hydroxyl radical release in the brain. This results in decreased serotonin and dopamine concentrations in the striatum [10].

### 1.2. Review of PEA: Stimulant Derivatives

2-Phenylethylamine derivatives can be categorized into two groups: stimulants and hallucinogens. Stimulants are drugs that increase the activity of the central nervous system. These substances are frequently used in the treatment of ADHD, most commonly in the form of derivatives such as methylphenidate and amphetamine [15,16]. Both drugs take effect approximately 30 to 45 min after ingestion. Amphetamines, however, act longer—ranging from 4 to 16 h depending on the preparation—whereas methylphenidate lasts up to 12 h. Methylphenidate is also employed in the treatment of apathy in patients diagnosed with Alzheimer’s disease [17]. Preliminary studies [18] have indicated that patients treated with methylphenidate exhibit a shift in their pain threshold. Furthermore, research has demonstrated that methylphenidate has the capacity to enhance the analgesic effects of morphine, thereby impacting the management of pain [18]. Both drugs modulate the activity of dopamine and norepinephrine in striatocortical regions. Methylphenidate functions as an allosteric inhibitor of dopamine and norepinephrine reuptake (by binding to their transporters), while amphetamines act as compensatory inhibitors. The reuptake inhibition that these drugs induce results in increased bioavailability of dopamine and norepinephrine. The abuse of these drugs results in the release of dopamine into the synaptic gap, leading to a sudden improvement in mood and the potential for the development of addiction [19,20]. The most prevalent cause of mortality resulting from amphetamine use, particularly methamphetamine, is cardiovascular dysfunction. This is attributable to several factors, including arterial narrowing, elevated blood pressure, the development of atherosclerotic plaques, and alterations in heart rhythm [21].

Methylenedioxyamphetamine (MDMA), also known as 3,4-methylene-dioxymethamphetamine (ecstasy), and 2,5-dimethoxy-4-methylamphetamine (DOM) are synthetic psychedelic amphetamines. MDMA is among the most commonly abused non-medical psychotropic substances [22,23]. Its excessive use has been associated with fatalities resulting from hepatic, cerebral, and cardiovascular toxicity, as well as serotonin syndrome. Clinical studies have demonstrated the efficacy of MDMA in the treatment of treatment-resistant post-traumatic stress disorder (PTSD). A similar mechanism of action has been observed in other amphetamine derivatives. These involve inhibition of serotonin, dopamine, and norepinephrine transporters, with particularly strong serotonergic effects. This leads to increased release of serotonin (responsible for its empathogenic and entactogenic properties), as well as dopamine and norepinephrine, into the synaptic gap [24,25]. This phenomenon is associated with a reduction in the activity of emotional memory pathways within the amygdala and insula regions. This leads to a reduction in feelings of fear, an increase in feelings of calmness and safety, and the reprocessing of traumatic memories. When these effects are combined with behavioral therapy, it has been observed to improve the mental condition of patients diagnosed with PTSD [26,27].

### 1.3. Review of PEA: Hallucinogenic Derivatives

Hallucinogens, also known as psychedelics, are substances that have been used for millennia by indigenous cultures for sacred and medicinal purposes. The use of mescaline has been documented as early as 5700 years ago. These substances attained widespread popularity during the 1960s. However, their use was subsequently outlawed during the 1970s [28,29]. The mechanism of action of these substances involves stimulation of the 5HT_2A/2C_ serotonin receptor. This leads to hallucinatory states, altered sensory perception of the environment, and experiences often described as spiritual. The use of hallucinogens in the therapeutic management of depressive, anxiety, obsessive-compulsive, and addiction-related disorders is predicated on their substantiated neuroplastic effects. The dendritogenic, synaptogenic, and neurogenic effects of hallucinogens have been demonstrated primarily in the prefrontal cortex and hippocampus. These regions support learning and increase synaptic strength in the reward system. Effects are associated with temporary improvements in mood, which can be made permanent under the right conditions of use [30]. A taxonomic classification of psychedelics reveals two primary groups of alkaloids. The first group consists of tryptamines, which include psilocybin and DMT. The second group consists of phenethylamine (PEA) derivatives, including mescaline and synthetic compounds. Stimulants derived from 2-phenylethylamine, such as 2,5-dimethoxy-4-methylamphetamine and MDMA, are also classified as hallucinogens [28]. Mescaline, a naturally occurring substance, is found in cacti, particularly in the peyote cactus (*Lophophora williamsii*). This compound is identified as a serotonin 5HT_2A_ receptor agonist. The effects of mescaline include dilation of the pupils, an increase in body temperature, and changes in blood pressure and heart rate. Mescaline has been observed to induce synesthesia. Typical visual impressions are kaleidoscopic, and at higher doses, perceptions of time and space become distorted. Adverse effects, such as anxiety, depression, and restlessness, have also been reported [31,32].

### 1.4. Review of Opioids

Opioids, such as methadone, tramadol, and fentanyl, are not 2-phenylethylamine derivatives but are included in this study due to their impact on psychomotor performance and driving safety. Opioids are the most established group of drugs employed for the management of severe pain, particularly in the context of postoperative and cancer-related pain. The term “opioid” encompasses both naturally occurring and synthetic or semi-synthetic drugs that mimic the effects of endogenous opioid systems by binding to their respective receptors [33,34].

**Figure 2:**
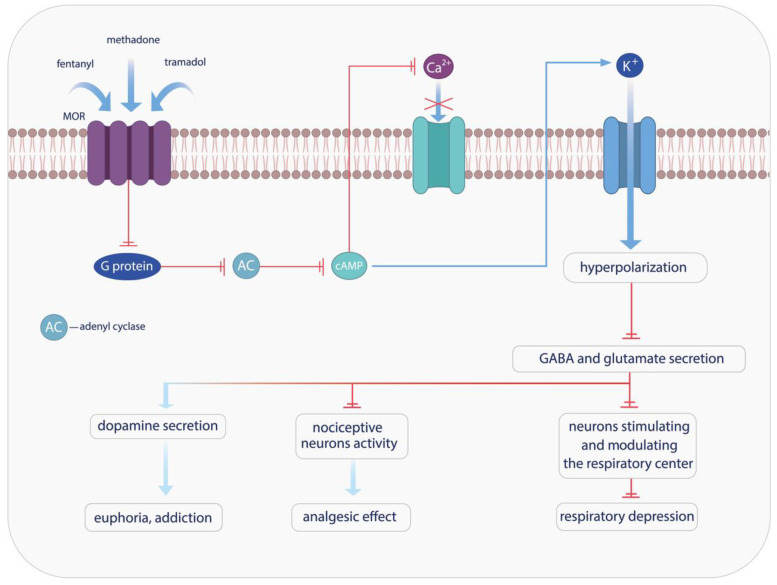
The effect of opioid-induced MOR receptors activation.

Studies have shown the presence of three opioid systems: pre-proenkephalin, pre-proopiomelanocortin, and pre-prodynorphin. There are also three types of μ (MOR), κ (KOR), and δ (DOR) receptors, which are localized and can be activated at all levels of peripheral and central nerves (Figure 2) [33,34,35]. Methadone, a synthetic opioid receptor agonist, has demonstrated efficacy in the treatment of pain and opioid dependence [33]. Methadone is often the first-choice opioid for managing pain resulting from cancer and renal dysfunction. It is selected when financial resources are limited due to its relatively low production cost [36]. However, methadone use carries potential risks, including respiratory depression and arrhythmias, which can lead to fatal outcomes. Most fatalities associated with this drug occur during sleep, caused by QT interval prolongation, which has been shown to increase the risk of potentially lethal torsade de pointes arrhythmia [21]. Tramadol exerts a multifaceted pharmacology, encompassing effects on the opioid, noradrenergic, and serotonergic neurotransmitter systems. Its use in the management of pain is comparable to that of methadone, yet it is prescribed more frequently, as it is associated with a reduced risk of respiratory depression and addiction [35]. The use of this agent has been demonstrated in the management of a variety of pain conditions, including post-operative pain, neuropathic pain, cancer-related pain, degenerative pain, perinatal pain, pain resulting from trauma, and renal or biliary colic. In addition, it has been demonstrated to enhance mood in cases of depressive and anxiety disorders [37,38]. Tramadol exerts its effects on both peripheral and central neurons. Two enantiomers, each exhibiting a distinct action that is complementary to the other, are involved in the augmentation of the analgesic effect. Importantly, both enantiomers function as reuptake inhibitors, with (−)-tramadol targeting norepinephrine and (+)-tramadol acting on serotonin [39]. The inhibition of these two compounds’ reuptake has been shown to induce specific adverse effects, including serotonin syndrome and seizures [40].

Fentanyl, a synthetic opioid, is the most commonly used agent for intraoperative anesthesia. Like other opioids, it is utilized for the management of pain related to various medical conditions, including cancer, postoperative pain, and myocardial infarction. Its potency is now considered the leading cause of death among adults aged 18 to 45 in the US and a growing public health problem in Europe [41]. The substance has minimal cardiovascular risk and does not increase histamine levels. Additionally, it is inexpensive to synthesize, a factor that has influenced its popularity [42,43]. In recent years, non-medical fentanyl use has increased. This trend has been accompanied by a significant rise in drug-related deaths. The phenomenon of fentanyl addiction is attributed to its engagement of the reward system, which subsequently stimulates the use of additional doses of the substance. Moreover, fentanyl use is often accompanied by withdrawal-induced discomfort, which subsides after the administration of subsequent doses of the drug [44].

Characterization of 2-phenylethylamine and its derivatives is concluded in Table 1.

### 1.5. Overview of DUID Problem

Accurately determining the scale of the issue addressed in this chapter is highly problematic, as it relies primarily on roadside surveys, which are voluntary. Individuals under the influence of substances impairing their psychomotor functions often refuse to participate. As a result, the data are frequently unrepresentative, leaving the true extent of the phenomenon uncertain. An example of this may be a study of taxi drivers involved in accidents, in which the concentration of psychoactive substances in their bodies was examined: out of 80 individuals potentially eligible to participate, 20 refused [45,46]. Some studies conducted on drivers involved in traffic accidents have shown that up to 54% were under the influence of alcohol and/or drugs, with 9.3% testing positive for opioids, 10.8% for stimulants, and 7.5% for sedative medications [47]. The statistics for taxi drivers involved in accidents are as follows: 53.3% were under the influence of psychoactive substances, with nearly one-third refusing to participate in the study [46].

Individuals who use these drugs are predominantly male [48]. It is also noteworthy that both cocaine and amphetamine-like substances exhibit a marked increase in the propensity for male individuals to operate motor vehicles while under the influence of these substances. This tendency is less pronounced in female individuals [49].

The following section provides an overview of the issue of driving under the influence of selected psychoactive substances.

### 1.6. DUID Problem: Methamphetamine (METH), 3,4-Methylenedioxymethamphetamine (MDMA) and Amphetamine

In the United States, it is estimated that one million cases of driving under the influence of methamphetamine and 3,4-methylenedioxymethamphetamine occur annually [50]. Amphetamines, even in low doses, have been shown to enhance cognitive functions such as focus and alertness [48]. Additionally, methamphetamine may increase both positive and negative mood states [51]. However, increasing doses or combining the drug with alcohol has the potential to counteract these effects, leading to reckless driving and impaired judgment. The effects of driving under the influence of amphetamines can vary, with some individuals experiencing significant agitation while others demonstrate increased calmness [48]. The propensity for driving under the influence of amphetamines in Victoria, Australia, suggests a heightened frequency of drug use during weekends and the transition from late-night to early-morning hours [52]. In Poland, based on wastewater analysis conducted in Poznań at the turn of 2009/2010 [53], it was concluded that the levels of amphetamine, methamphetamine, and MDMA consumption were lower than in other European countries. Moreover, the consumption of methamphetamine and MDMA was found to be higher than that of amphetamine. Periodic fluctuations were also observed, with temporary peaks in November, December, May, June, and July, which are associated with examination periods in high schools and universities [54].

As with amphetamine, MDMA has been shown to produce positive effects on specific psychomotor abilities, including faster reaction times and improved track maintenance. However, due to its more pronounced psychedelic effect, MDMA has been shown to have adverse effects on driving abilities. These include higher velocity and greater fluctuations over time. These adverse effects are particularly pronounced after the ingestion of larger doses [54,55]. The present study seeks to establish a correlation between the side effects of stimulants belonging to the amphetamine group, particularly MDMA, and their strong sympathomimetic properties, as well as the occurrence of serotonin syndrome. Consequently, this may result in cardiac damage, including valve dysfunction. Furthermore, MDMA use may cause a sudden drop in blood pressure and acute hyponatremia. This, in turn, leads to osmotic imbalance, muscle spasms, and convulsions [56,57,58]. An increase in negative mood has also been observed following MDMA consumption [51]. 3,4-Methylenedioxymethamphetamine is increasingly used by adolescents in Europe. In a cross-sectional study conducted among medical students in France (using an online questionnaire), it was found that 21.5% of the respondents had experimented with MDMA. Additionally, studies indicate that most of them are exposed to these substances at various parties and festivals [59]. A cohort study conducted in Canada among youth aged 14–26 who use drugs via injection showed that 63.4% had used MDMA in the past [60].

### 1.7. DUID Problem: Methylphenidate

In the United States, ADHD affects approximately 6% of the population [4]. In the absence of medication, the disease has been shown to contribute to decreased safety and more frequent driving errors. Situations involving low attention demands, such as highway driving, pose an increased risk of accidents, as patients often exhibit symptoms of inattention and impatience. The administration of medication has been demonstrated to markedly enhance driving performance [61]. Methylphenidate, one of the most commonly used treatments, has been shown to improve quality of life and academic performance while reducing the frequency of injuries and accidents [62]. Studies on individuals diagnosed with ADHD who were treated with methylphenidate demonstrated that these subjects exhibited faster lane departure responses, more precise speed maintenance, and superior brake control [63]. Methylphenidate enhances concentration, making it a potentially desirable substance not only for individuals diagnosed with ADHD but also for students without the disorder. A study conducted among medical students in South Africa found that 28.1% had used methylphenidate [64]. A meta-analysis of 15 randomized controlled trials by Gobbo and Louzã [65] confirmed that methylphenidate, particularly the osmotic-controlled oral system (MPH-OROS) and immediate-release (MPH-IR) formulations, consistently improves driving performance in ADHD patients, especially teenagers and young adults, compared to placebo or no-drug conditions [65].

### 1.8. DUID Problem: Opioids

#### 1.8.1. Overview of Fentanyl

Since 2010, due to increasing popularity and availability, fentanyl has been increasingly detected in DUID cases [66]. In light of the growing trend, it is worth noting the drug’s impact on psychomotor function and driving skills [67]. The manner in which fentanyl is administered can exert a substantial influence on an individual’s driving capacity, as highlighted in the meta-analysis conducted by Rohrig et al. [68]. In their study, Rohrig et al. examined the impact of fentanyl on driving ability in 21 patients. After three months of opioid therapy, low doses of fentanyl appeared to have minimal, almost negligible, effects on driving ability. In a parallel study [68], 21 patients receiving consistent doses of fentanyl patches for a minimum of 14 days exhibited no substantial deviations in psychomotor test outcomes compared to the control group [68]. Research findings suggest that stable doses of transdermal fentanyl may have a limited impact on driving performance [67]. Furthermore, the alleviation of chronic pain has been demonstrated to exert a favorable influence on the patient’s quality of life. Improvements in attentiveness, visual memory, and hand-eye coordination have been observed [69]. In a separate study, nine volunteers with no history of exposure to opioids were administered 100 µg of fentanyl intravenously. Symptoms such as psychomotor impairment and prolonged reaction time were observed in these volunteers even two hours after the drug was administered. The results of psychomotor tests conducted on nine other healthy volunteers revealed that fentanyl concentrations exceeding 2.5 ng/mL resulted in a 15–30% reduction in performance relative to baseline values [68]. Increased tolerance from repeated use allows fentanyl to be maintained in the blood at concentrations higher than the standard therapeutic range [70].

#### 1.8.2. Overview of Methadone

The findings from studies conducted on methadone-treated patients demonstrate that subjects under the influence made faster decisions in cognitive-motor tests, but they were more prone to errors [71]. Research has demonstrated that patients who are prescribed methadone and who also abuse opioids exhibit deficits in short-term working memory [72].

#### 1.8.3. Overview of Tramadol

Patients with chronic non-cancer pain treated with tramadol show slightly poorer lane-keeping ability compared with healthy control groups. However, the results of a large proportion of the subjects were considered safe according to the criteria. The effect of the tests was attributed not only to the use of tramadol itself, but also to the presence of chronic pain [73].

Opioid use has been associated with a wide spectrum of adverse effects, ranging from addiction to psychomotor disorders that can impair driving ability. The occurrence of side effects subsequent to the ingestion of low concentrations of opioids relevant to driving can manifest as sedation, dizziness, and drowsiness; these symptoms frequently dissipate over the course of long-term therapy [74]. The effects of opioids on psychomotor abilities, driving performance, and road safety require further research and careful individual assessment [68].

### 1.9. DUID Problem: Hallucinogens

According to a meta-analysis by Salas-Wright et al., [75] approximately 10% of hallucinogen users in the United States report driving under the influence of hallucinogens (DUIH). Use of these drugs (e.g., mescaline) leads to psychomotor and perceptual impairment, thereby reducing driving safety and increasing the frequency of crashes. The study, which was conducted on 4447 individuals between the ages of 16 and 64, found that non-Hispanic Black/African American respondents were more likely to drive under the influence of hallucinogens than representatives of other racial/ethnic groups (16% vs. 8%). Moreover, individuals under the influence of these substances exhibited a heightened propensity for alcohol and drug abuse. Among respondents reporting DUIH, in addition to hallucinogens, 80% also reported driving after using marijuana, which exacerbates impaired driving control and increases the risk of dangerous behavior [75].

No additional studies describing the specific effects of mescaline on psychomotor function and driving were identified in the literature that met the inclusion criteria.

In their meta-analysis, Tirri et al. [76] demonstrated that 25H-NBOMe [2-(4-iodo-2,5-dimethoxyphenyl)-N-(2-methoxybenzyl)ethanamine] and its halogen derivatives, 25I-NBOMe [2-(4-iodo-2,5-dimethoxyphenyl)-N-(2-methoxybenzyl)ethanamine] and 25B-NBOMe [2-(4-bromo-2,5-dimethoxyphenyl)-N-(2-methoxybenzyl)ethanamine] exhibit stronger psychoactive effects than their 2C analogs and LSD [76].

### 1.10. Definition and Importance of Psychomotor Functions

**Figure 3:**
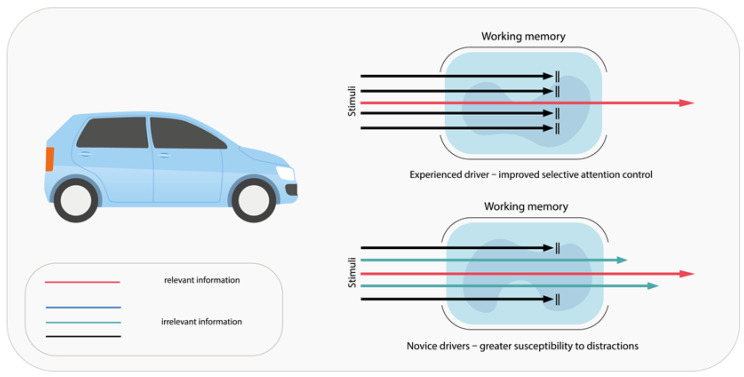
Schematic representation of the association between drivers’ resistance to distraction and their driving experience, attributed to the enhanced ability to efficiently filter out irrelevant information.

Cognitive functions are organized hierarchically, from basic sensation and perception to more complex processes requiring the coordination of multiple stimuli. Sensory information is gathered through the human senses and then identified and categorized as perception. Attention and concentration are among the fundamental pillars of cognitive function. A disruption of these components gives rise to what is termed “selective attention,” a critical facet of driving activities. This process entails the ability to discern pertinent information while effectively filtering out extraneous stimuli. It has been demonstrated that experienced drivers exhibit a greater tolerance for distractions when driving than do novice drivers [77] (Figure 3).

Attention, defined as the ability to be aware of both internal information (e.g., one’s own thoughts) and the external environment, is an important element. Research has indicated a positive correlation between attention and an increase in safety behaviors, a reduction in cognitive errors and violations of safety procedures, positive responses to risk, concentration, self-control, and better adaptive responses to stress [78]. Another cognitive domain that is pertinent to driving is the speed of information processing, which is defined as the rate of analysis of incoming stimuli [79]. The fluidity and integrity of cognitive functions are essential for optimal driving safety. Impaired visual perception hinders the accurate assessment of road conditions, while executive dysfunction impedes timely responses in emergency and hazardous situations. Memory disorders hinder the utilization of previously acquired skills and experiences [80]. This issue is of critical importance due to the significant number of traffic accidents caused by driver distraction; it is estimated that one in five drivers fails to pay adequate attention to the road while operating a vehicle [81].

**Figure 4:**
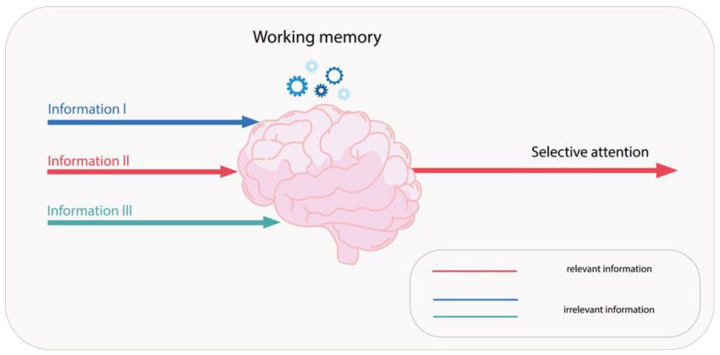
Working memory enables the selection of incoming stimuli, leading to selective focus on the most relevant information.

The role of working memory in the information processing of drivers is a pivotal one [82]. It has an individually determined capacity, temporarily stores and modulates information, and filters signals by focusing attention on relevant stimuli (Figure 4) [83].

Working memory capacity (WMC) is defined as the amount of information an individual can retain and manipulate within their immediate cognitive scope. Research findings indicate that the level of WMC is inversely related to the likelihood of making errors. Furthermore, the results of a subsequent study corroborate the positive correlation between WMC and the capacity to detect them. This correlation is characterized by a reduction in the time required to obtain a response and an enhancement in its precision [79]. As previously noted, driver distraction accounts for many traffic accidents. Age is directly related to the development of working memory capacity. The anatomical bases of this relationship are located in the prefrontal cortex (PFC) and the parietal lobes, which undergo a period of maturation beginning around the age of 11 years and persisting until approximately 25 years of age [84]. Automated activities are defined as secondary processes that occur alongside the primary reaction. They do not compromise its efficiency. The process of automation of processes that initially required control occurs over time [77]. Therefore, another reason for the reduced resistance to side factors of younger drivers is their shorter seniority [84].

**Figure 5:**
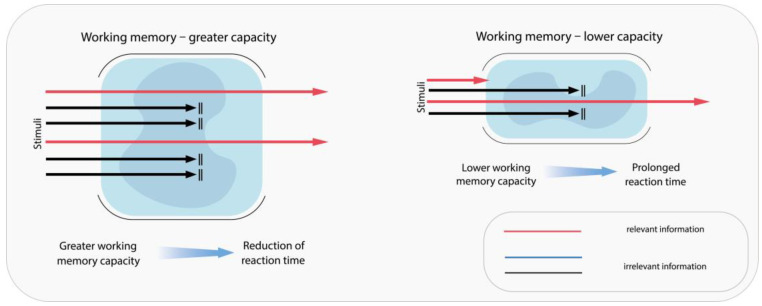
Schematic representation of the hypothesis demonstrating a positive correlation between working memory capacity and reaction time.

Higher WMC is associated with enhanced cognitive coordination and improved safety outcomes. Examples include the ability to maintain lane position and the physiological response of pupil dilation upon threat detection, both of which serve to decrease reaction time (Figure 5). Drivers with higher WMC have been shown to exhibit higher levels of concentration, faster heart rates, lower heart rate variability, and lower levels of oxyhemoglobin (OxyHb) and deoxyhemoglobin (DeoxyHb).

Furthermore, studies have demonstrated that cognitive working memory training has quantifiable impacts on enhancing driving proficiency [78]. A critical parameter that must be addressed in any discussion of psychomotor functions in the context of driving is reaction time. The term is defined as the temporal interval from the initial perception of danger to the initiation of a defensive reaction by the operator of the vehicle. It is subject to fluctuations that are contingent not only on the mental state of the driver (e.g., stress) or their physical state (e.g., fatigue), but also on their level of experience. There are three components of reaction time:

− perception time-the period from the appearance of a threat in the driver’s field of vision to the moment when attention is drawn to it and it is recognized;
− basic psychological reaction time-this is the period of analyzing an event and making a decision, e.g., whether to brake or to avoid an obstacle. It also includes sending a signal to the motoneurons to initiate a maneuver;
− foot transfer time—the time associated with a reaction, such as transferring the foot from the accelerator to the brake pedal.

**Figure 6:**
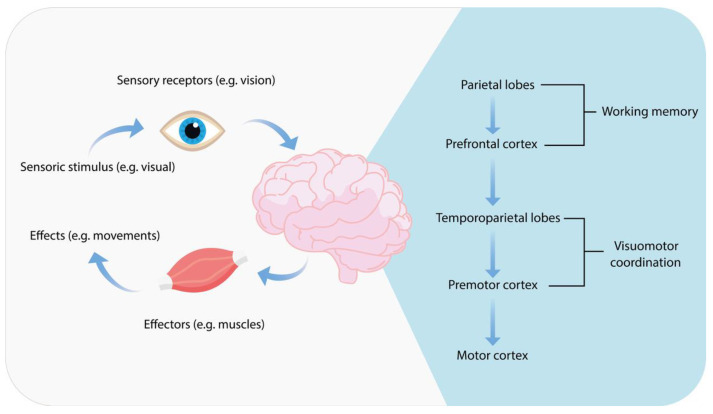
Schematic correlation between a sensory stimulus, its transformation through working memory, and its motor interpretation, briefly illustrating its neural pathway in the brain.

Reaction time is influenced by the complexity and predictability of the stimulus—responses to simple and expected stimuli require less time [85]. Sensory processes, including the reception of somatosensory stimuli, initiation of motor responses, and regulation of actions, are central to the driving process [86]. Visual-motor coordination can be defined as the process of converting an object’s visual localization into its corresponding motor coordinates [87]. Such a complex action has been shown to activate the temporal-parietal-occipital regions and the premotor area, which are responsible for generating appropriate motor responses (Figure 6) [86].

This process demonstrates adaptability, operating on the principle of learning to adjust motor precision to align with visual stimuli and achieve the intended goal. The foundation of the adaptation process is short-term changes in neuronal activation patterns, leading to behavioral policy adjustments [88]. The efficacy of eye-motor coordination hinges upon temporal analysis, which plays a pivotal role in predicting the location of a moving object when the hand reaches it, based on its position when the motor response is initiated. Impairments in this process, with delays of up to 40 milliseconds, have been shown to disorganize the visual behavior involved in tracking fast-moving objects. Delays of a second or more can disrupt the ability to interact effectively with the environment [87].

### 1.11. Current Regulations on Driving Under the Influence of Selected Substances in Poland

In Poland, the legal situation regarding operating a vehicle under the influence of medications is quite complex. This results primarily from the possibility of interpreting legal regulations, which in turn arises from the ambiguity of the wording used to describe specific provisions. According to Article 178a of the Penal Code, operating a vehicle (mechanical, in land, water, or air traffic) while intoxicated or under the influence of an intoxicating substance may result in a penalty of imprisonment for up to three years [88]. According to this provision, Polish law prohibits driving a vehicle after exposure to substances referred to in the regulation as broadly defined “intoxicating” agents. A list of such substances can be found in the Single Convention on Narcotic Drugs of 1961 [89,90]. However, it does not include any of the medications discussed herein, with the exception of methadone. This is not the only legal regulation on the matter–the Regulation of the Minister of Health on the list of psychotropic substances, narcotic drugs, and new psychoactive substances–classifies the aforementioned medications, if their concentration exceeds the established threshold value (LOQ), as follows:

− Group I-P psychotropic substances–MDMA;
− Group II-P psychotropic substances–methylphenidate and amphetamine;
− Group I-N narcotic drugs-Fentanyl and methadone [90].

Not all medications are classified as narcotic drugs, so does this mean that one may drive under their influence? This question is best addressed by the Supreme Court Resolution of 27 February 2007, file no. I KZP 36/06, which states “The term intoxicating substance within the meaning of Article 178a of the Penal Code includes not only the intoxicating substances listed in the Act of 29 July 2005 on Counteracting Drug Addiction (Journal of Laws No. 179, item 1485), but also other substances of natural or synthetic origin that act on the central nervous system and whose use decreases the ability to operate a vehicle” [91]. According to this information, any substance with a proven effect on the central nervous system (CNS) that may negatively affect the ability to drive is treated as an intoxicating substance. Thus, it may be concluded that all substances listed in the previously mentioned Regulation of the Minister of Health are potentially dangerous in the context of driving motor vehicles.

At this point, it is important to emphasize the role of the physician, who, in accordance with Article 31 of the Act of 5 December 1996 on the Professions of Physician and Dentist, is obliged to provide the patient or their legal representative with accessible information about their health condition, diagnosis, proposed and possible diagnostic and therapeutic methods, the foreseeable consequences of their application or omission, treatment results, and prognosis. This obligation includes informing the patient about the potential effects of certain medications on psychomotor functions and the resulting impairments, including the ability to drive a vehicle [92].

Based on the provisions presented above, a patient taking medications that affect psychomotor efficiency, who is aware of their effects, is fully responsible for their actions. Legal consequences depend on the type of incident:

− Operating a vehicle under the influence of an intoxicating substance carries a penalty of imprisonment for up to three years [88];
− Causing an accident in which another person sustained bodily injury is punishable by up to 3 years of imprisonment, or, in the case of serious injury or death, the perpetrator may be sentenced to imprisonment from six months to eight years [88,92].

## 2. Materials and Methods

### 2.1. Literature Research

This systematic review was carried out with the Preferred Reporting Items for Systematic Reviews and Meta-Analyses (PRISMA) 2020 guidelines (see Supplementary Materials to consult the PRISMA Checklist, Tables S1 and S2) [93]. A systematic literature search was conducted on 17 June 2025. Relevant publications were identified by searching the electronic databases: PubMed, Embase, and Web of Science, applying expanded search terms for articles published from January 2000 to the end of May 2025 (see the Supplementary Materials to consult the Systematic research list). The full search strings used for each database are available in the Supplementary Materials. This timeframe was chosen due to advancing technology and development of 2-phenylethylamine derivative drugs. In addition, bibliographies of identified articles were searched for relevant studies.

Studies were included if they reported the following inclusion criteria: study performed on adult humans, included vehicle driving ability and use of 2-phenylethylamine derivatives or selected opioids, empirical study with available full text in English. Articles not providing English full text, not mentioning driving ability and 2-phenylethylamine derivatives or selected opioids usage or studies performed on populations below 18 years old were excluded.

Two independent reviewers screened titles and abstracts. Each full-text article was evaluated by two other independent researchers. Any disagreements at either stage were resolved by discussion or adjudicated by a third author. Screening and data extraction were performed using Rayyan [92].

### 2.2. PICO Framework and Risk of Bias Assessment

The PICO method was implemented for data extraction, with the following protocol:

- Patient: adults taking selected 2-phenylethylamine derivatives (2-phenylethylamine, L-phenylalanine, phenylacetic acid, methylphenidate, amphetamine, methamphetamine (METH), mescaline, methylenedioxyamphetamine (3,4-methylenedioxymethamphetamine, MDMA, ecstasy), 2,5-dimethoxy-4-methylamphetamine (DOM) or selected opioids (methadone, tramadol, fentanyl).
- Intervention: exposure to selected 2-phenylethylamine derivatives or opioids.
- Comparison: population not taking 2-phenylethylamine derivatives or opioids, placebo, the same population with and without 2-phenylethylamine derivatives or opioids.
- Outcomes: changes in psychomotor functioning, affect, cognitive impairment, influence on driving safety, reaction time, engaging in reckless driving, lane-keeping ability.

This PICO framework directly guided study eligibility and structured data extraction.

Additionally, other data extracted included patient condition (e.g., ADHD diagnosis), route of drug administration, and study design. Prior to synthesis, extracted outcome data were organized into structured tables, and studies were grouped by individual substances and synthesized narratively according to psychomotor and cognitive outcomes. The synthesis process included categorization of outcomes into domains related to psychomotor performance and cognitive function. All results that were compatible with each outcome domain in each study were sought. The risk of bias was assessed using RoB2 protocol by two independent researchers for each study.

Overall, some of included studies were judged to have moderate concerns, mostly because of risk of bias due to measurement of the outcome. 17 studies were judged at low risk of bias, while 14 studies were judged at moderate risk. Full details are provided in Table S4 in the Supplementary Materials.

No sensitivity analyses were conducted due to the narrative nature of the synthesis, and certainty of evidence was not formally assessed due to the heterogeneity of the included studies and the non-quantitative nature of the synthesis. Effect direction and significance were extracted narratively. No quantitative synthesis was performed.

## 3. Results

**Figure 7:**
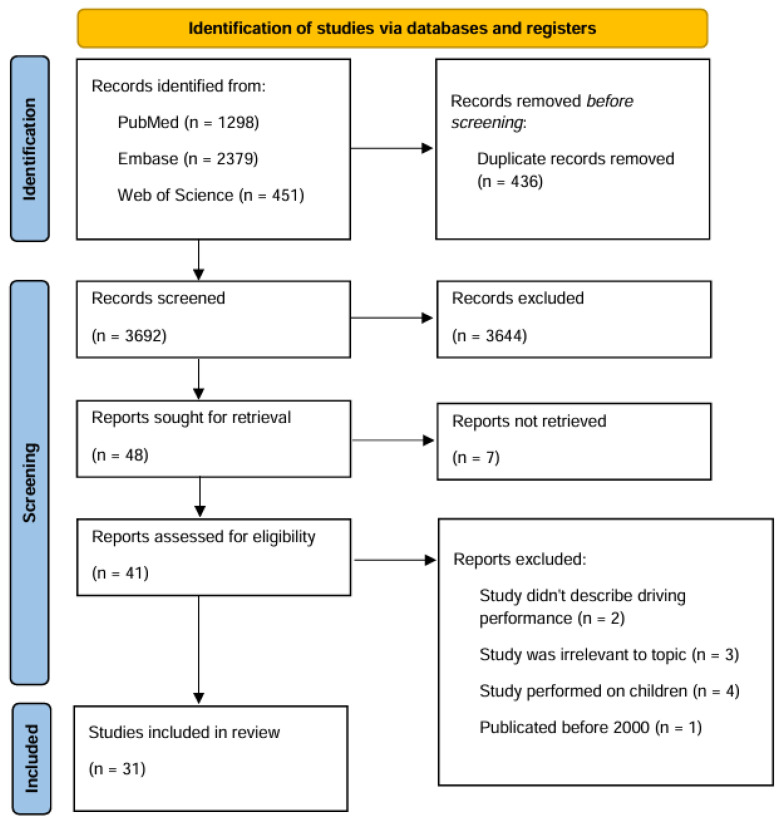
Flow diagram of literature search based on PRISMA guidelines (n = number of scientific articles; time frame: January 2000 to the end of May 2025; database: PubMed, Embase and Web of Science).

A total of 4128 articles were identified, and after resolving duplicates, the search identified 3692 unique articles. Two independent researchers screened these articles and, after resolving conflicts, 49 articles were selected by title and abstract, from which 31 articles were deemed as included by another three independent researchers after full text evaluation (Figure 7).

The included studies were grouped by investigated substance:

Amphetamine (n = 4)

Methamphetamine (n = 5)

Methylphenidate (n = 7)

MDMA (n = 6)

Fentanyl (n = 3)

Methadone (n = 7)

Other (n = 1)

Two of the included studies were assigned to two groups due to investigating more than one substance of interest. None of the studies regarding mescaline, tramadol, and 2,5-dimethoxy-4-methylamphetamine (DOM) met the inclusion criteria.

Selected studies focused on adult participants (healthy or with clinical conditions such as ADHD or chronic pain) and assessed driving performance and psychomotor function with exposure to phenylethylamine derivatives. Study designs included randomized controlled trials, crossover trials, observational studies, and simulator-based experimental designs. The characteristics of each study are summarized in Table S3 in the Supplementary Materials.

During full-text screening, 18 studies were excluded for the following reasons:

No full-text available (n = 8);

Study did not describe driving performance (n = 3);

Observational study describing drug usage in accident victims (n = 3);

Population below 18 years old (n = 2);

Publication before 2000 (n = 1);

Irrelevant drug (n = 1).

Each study’s risk of bias was assessed using the RoB 2 scale [85] by two independent researchers and summarized in Table S4 of the Supplementary Materials.

### 3.1. Results: Amphetamine

Studies on amphetamine showed that in low therapeutic doses amphetamine may improve driving performance both in healthy population and population with ADHD, indicated by improved attention, faster reaction time and better lane keeping. The effects improving driving are observed quickly after administration, but over time and with a decrease in blood concentration, there is a deterioration in psychomotor skills, which can significantly increase the risk of accidents. Amphetamine, like methylphenidate, has been shown to enhance driving performance in patients diagnosed with ADHD. However, its mechanism of action is more extensive and carries a higher risk of abuse when compared with other stimulant drugs. Unlike methamphetamine, the effects of amphetamine are shorter and usually less intense, which partially limits its negative impact in experimental conditions [94,95,96,97,98,99]. However, higher recreational doses may have no impact or negative effect on overall driving performance [96,99].

### 3.2. Results: Methamphetamine

Methamphetamine demonstrated stimulating effects on attention and alertness in most studies [52,98,100,101], but these were negated by deteriorated executive control and erratic driving behaviors, with these effects being more significant at higher doses. Experimental studies showed it may increase speed variability, poor lane control, and agitation or overconfidence, often accompanied by mood swings. Of note, one experiment reported no effect of methamphetamine on driving performance 2–3 h post-dosing [98], but this finding should be interpreted with caution. This result needs to be weighed against the established pharmacokinetic profile of the drug, such as a relatively fast onset followed by a gradual decline from the bloodstream. At this time interval, plasma levels of the drug would be practically at C_max_, and, hence, notable pharmacological effects would be expected. As definite impairments in driving performance and cognition have been reported by most other studies, this single finding should not be viewed as equal evidence but as a potential anomaly based on methodology differences, small sample size, or measurement specificity. Therefore, further research is needed to validate the consistency of these findings with the well-known pharmacokinetic profile of methamphetamine and with outcomes from other studies.

### 3.3. Results: Methylphenidate

Most studies focus on populations diagnosed with ADHD [63,102,103,104,105,106], with one study including healthy individuals [107]. These studies unanimously demonstrate that methylphenidate improves driving performance in this population across all measured parameters. Outcomes included faster brake response, improved lane keeping, and better speed regulation. Additionally, no relevant adverse effects on cognition or psychomotor skills were observed at therapeutic doses. Improvements were particularly significant during low-stimulation driving tasks (e.g., highway driving), where inattentiveness is usually most pronounced. The improvement in driving is more pronounced in people with ADHD, in whom the drug normalizes attention deficits, while in healthy individuals the effects are moderate or minimal. This suggests that the beneficial effects of methylphenidate depend on the baseline level of cognitive performance. It is worth noting that, compared to amphetamine, methylphenidate has a milder profile of action and a lower risk of abuse, while showing similar effectiveness in improving psychomotor skills in patients with ADHD.

### 3.4. Results: MDMA

MDMA was found to increase speed variability, risk-taking, and reduce vehicle control in a few studies [55,101], with rest of the studies stating that it has no effect on driving performance [106,107,108,109,110,111]. In comparison to classic stimulants such as amphetamine or methamphetamine, MDMA has a more pronounced effect on emotions and perception. An increase in serotonin can result in disturbances in the perception of time and space, and dopaminergic stimulation can lead to excessive self-confidence and an increased propensity for risk-taking. MDMA does not invariably lead to evident psychomotor deficits. This discrepancy may serve to elucidate the observed variations in the results of experimental studies. It should be noted that, as a party drug, MDMA is often consumed with other drugs–MDMA in combination with alcohol showed a significant reduction in driving performance [55,110].

### 3.5. Results: Fentanyl

Studies [69,112,113] show that acute administration of fentanyl in low doses (25 μg) may have limited effects on driving performance. This dose can be increased to 50 μg in patients treated chronically. A 50 μg fentanyl injection in opioid-naïve patients caused significant reaction time delays, reduced motor accuracy, and symptoms of sedation. In higher doses (100 μg), fentanyl impairs driving speed and precision for up to 120 min post-administration in both chronic users and people not treated. Due to its fast metabolism, the impairing effects are negligible after two to three hours post-administration. However, these effects are significantly less pronounced in long-term users [113]. Chronic administration of opioids (such as fentanyl) leads to the development of tolerance. This denotes the necessity of administering progressively higher doses of the drugs in order to achieve the same opioid effect, since brain cells possessing opioid receptors become less sensitive [114]. Fentanyl acts, among others, on µ-opioid receptors [115]. The precise mechanisms of fentanyl’s influence on neuronal activity in the brain, which lead to persistent alterations in cognitive functions and behavior, remain poorly understood [116]. The development of tolerance and decreased sensitivity is likely mediated by a mechanism involving the uncoupling of opioid receptors from G-proteins, with which they are normally associated. This process prevents G-proteins from exchanging GDP for GTP [117]. Fentanyl exhibits a specific pattern of action, in addition to its effects on MOR, it also displays affinity for certain types of adrenergic receptors. Such a mechanism may underlie the unique pathophysiology of this compound’s effects compared with other opioids [115]. Many molecular adaptations occurring in the brain under the influence of fentanyl remain unexplained. Studies indicate that chronic administration of fentanyl in mice leads to brain changes, including in the nucleus accumbens, a region involved in reward processing. These changes involve an increase in the number of synapses, widening of the synaptic cleft, as well as mitochondrial swelling and even rupture. It is believed that such permanent changes within the reward system may have a lasting impact on behavior [118]. In contrast, in an in vitro brain-on-a-chip study conducted in 2024 on mouse cells, it was demonstrated that acute exposure to fentanyl (100 nM) slightly decreased the frequency of action potentials and caused adaptation of firing rates in striatal medium spiny neurons (MSN) expressing the dopamine type 2 receptor (D_2_). Long-term exposure to fentanyl may inhibit the activity of striatal MSN through a non-opioid receptor–dependent pathway, probably modulated by cells expressing α1-adrenergic receptors [118].

### 3.6. Results: Methadone

Five studies [119,120,121,122,123] agree that methadone treatment significantly impairs driving and cognitive abilities, with an increased feeling of sleepiness. A wide, national study [119] reveals that exposure to methadone is associated with an increased risk of involvement in road traffic accidents resulting in bodily injury. One study performed on chronic methadone patients [124] found no significant differences in driving performance between treatment and control groups. One study performed on healthy individuals was concluded with a similar outcome [48]. It is noteworthy that in experimental studies on healthy individuals, the deficits were less pronounced than in patients undergoing chronic treatment. Methadone has a slow onset of action and a longer duration, which means that the disorders may persist for many hours after administration. The potential risks associated with early sedation extend beyond the immediate phase to encompass prolonged impairment of concentration.

### 3.7. Other Findings

An observational study performed by Brubacher [124], investigated drivers of on-road vehicles within six hours after a crash. The study found a positive correlation between psychoactive substance use (e.g., stimulants, opioids) and accidents while driving. Psychoactive substances were found in 56% of male drivers and in 52.4% of female drivers, with opiates being most prevalent–they were detected in as many as 10.9% of drivers [125]. Findings of the studies are presented in Table 2.

## 4. Discussion

Our review of the studies indicates the complexity of the impact of opioids and 2-phenylethylamine derivatives on driving ability and highlights the importance of further research to better understand the effects of this substances on psychomotor functions. The review of existing literature reveals significant heterogeneity of studies—covering diverse patient populations (e.g., healthy individuals vs. people with ADHD or chronic pain), different doses (therapeutic vs. recreational), routes of administration (oral, transdermal, intravenous), measured indicators (e.g., reaction time, lane-keeping accuracy, speed variability), as well as individual physiological parameters. These factors hinder quantitative analyses and the formal assessment of the reliability of evidence. For example, differences between populations—such as patients with ADHD, who may benefit from the use of methylphenidate, versus recreational users of MDMA or methamphetamine, who display marked impairment of psychomotor functions—make extrapolation of results between groups difficult. Similarly, variability in routes of administration—e.g., transdermal fentanyl in chronically treated patients vs. intravenous administration in opioid-naïve individuals—changes the pharmacokinetic profile and its related psychomotor effects, complicating the standardization of outcomes. We have attempted to address the issue of legal regulations in our country concerning driving under the influence of substances whose intake may potentially impair psychomotor performance. Clarity in these regulations will not be achieved until a universal method for testing such substances and their effects on humans is established.

It has been shown that opioids (methadone, tramadol, fentanyl), PEA derivatives including stimulants (amphetamine, methylphenidate) and hallucinogens (MDMA, mescaline), exert varying effects on drivers’ abilities. Stimulants used therapeutically and in appropriate doses—e.g., methylphenidate in patients with ADHD—may improve road safety by increasing coordination and reaction time during lane changes, enhancing precision and stability of maintained speed, as well as shortening braking time and improving safety. At higher doses or with recreational, clinically unregulated use of methylphenidate and its derivatives, the effect is neutral or even negative, leading to deterioration of psychophysical state and increased tendency toward dangerous, reckless driving behavior. A similar situation applies to amphetamine, where low therapeutic doses may enhance driving performance both in healthy individuals and in those with ADHD. In contrast, the use of methamphetamine does not yield beneficial effects, as the theoretically positive impact of increased attention and alertness is counterbalanced by impaired executive control and a heightened propensity for risky behaviors. The noradrenergic activity of amphetamine derivatives—e.g., the use of MDMA (ecstasy)—is associated with an increased risk of cardiovascular incidents in users. Methadone treatment is associated with impaired driving ability, increased drowsiness, and consequently a higher risk of traffic accidents. The use of hallucinogenic PEA derivatives, such as mescaline, likely reduces road safety, though reliable data examining the impact of these substances on psychomotor functions in the context of vehicle operation are lacking. Future research in this area should focus on describing the relationship between intake of hallucinogenic 2-phenylethylamine derivatives and psychomotor coordination in the context of operating motor vehicles, which would fill an important knowledge gap.

Recreational use of opioids, such as fentanyl, in unsupervised conditions, shows a harmful impact on perception and psychomotor performance—manifested as prolonged reaction time and impaired decision-making ability in drivers. Therapeutic doses of fentanyl administered chronically via the transdermal route do not impair driving ability in stable patients with developed opioid tolerance. However, individuals without prior opioid exposure, who have not developed tolerance, may experience acute impairment of psychomotor functions. Tramadol, in turn, may induce side effects such as sedation, dizziness, and drowsiness. Nevertheless, patients with chronic pain treated with tramadol demonstrate only slightly reduced lane-keeping performance, with outcomes often considered safe, which may be attributable to the presence of pain itself.

This systematic review has several limitations. One limitation of experimental studies is often the use of driving simulators, which unfortunately are not able to fully reflect real driving conditions, potentially reducing the accuracy of results. Secondly, the inability to precisely determine the composition and concentrations of psychoactive substances in recreational use makes it difficult to replicate actual blood levels in study participants and the physiological state under the influence of a given substance. Difficulties in reproducing research conditions and the growing popularity of synthetic recreational psychoactive substances emphasize the need to implement more advanced research methods based on standardized protocols—including uniform assessment criteria (e.g., validated indices in driving simulators), consistent dosing regimens, and precisely defined patient cohorts—which would increase comparability of results. Additionally, the included studies exhibited substantial heterogeneity in terms of study populations (healthy volunteers vs. patients with ADHD or chronic pain), investigated substances (stimulants, hallucinogens, and opioids), study designs (experimental, observational, simulator-based), and outcome measures (psychomotor tests, cognitive assessments, driving performance). This heterogeneity precluded quantitative synthesis and makes cross-study comparisons challenging.

We conducted risk of bias assessment only on the level of individual studies using the RoB2 scale, however we did not conduct a formal assessment of publication bias, and it is possible that studies with null or negative findings remain underrepresented in the literature.

We did not apply the GRADE approach to formally assess the certainty of evidence, and as a consequence, the overall confidence in the findings remains limited, and the conclusions should be interpreted with caution. Future research should address these issues by using standardized outcome measures, transparent reporting, and prospective preregistration.

Promising research directions include the integration of high-fidelity driving simulators with neuroimaging techniques, such as functional magnetic resonance imaging (fMRI) and electroencephalography (EEG), to study neural centers whose functioning is disrupted by 2-phenylethylamine derivatives and opioids. Pharmacogenetic studies could further explain individual differences in drug response, enabling personalized risk assessment related to driving and more precise therapy selection. The development of standardized biomarker panels to detect the presence and concentration of multiple psychoactive substances in biological samples could further increase detection accuracy and strengthen the correlation between substance presence and driving performance outcomes.

Advanced methodologies, such as adaptive driving simulators reproducing real-world road conditions or pharmacokinetic modeling that accounts for differences in routes of administration, may further enhance precision of results. Such an approach would allow for more accurate recreation of real driving scenarios and deeper understanding of the qualitative and quantitative effects of specific substances on drivers’ psychomotor functions. At present, there are no data specifying at what blood concentration the transition occurs from therapeutic to harmful dose. Solving these problems will facilitate the formulation of more reliable conclusions about the impact of psychoactive substances on road safety and provide the basis for developing regulations and clinical guidelines based on scientific evidence.

The main objective of future research should be to precisely determine the dose and long-term effects of 2-phenylethylamine derivatives and opioids. This information is essential for introducing changes in traffic and medical law that will increase driver safety.

## 5. Conclusions

Driving requires precise coordination of various psychomotor functions, including reaction time, motor coordination, and decision-making ability. Psychoactive substances, particularly 2-phenylethylamine (PEA) derivatives and opioids, may significantly affect these skills, and thereby road safety—depending on the type and dose of the substance. In summary, opioids—methadone, fentanyl, and tramadol—and PEA derivatives such as amphetamine or MDMA generally reduce psychomotor performance after intake, especially in high, non-clinically supervised doses, which translates into impaired driving ability in individuals under their influence, thus reducing road safety. Extending our conclusions further, it can be stated that such individuals may pose a serious threat when participating in road traffic. The results of this review, indicating improved driving ability in ADHD patients treated with therapeutic methylphenidate (thanks to improved reaction time, lane-keeping, and speed control), are consistent with the observations of Gobbo and Louzã [65]. They also align with the ADHD literature, confirming that controlled use of stimulants reduces driving deficits in untreated patients.

Current regulations regarding driving under the influence of psychoactive substances point to the necessity of careful management of pharmacotherapy that may affect driving ability. The results presented in this review highlight the need for meticulous management of these substances in the context of road safety and the importance of further research into their dose-dependent effects.

## Figures and Tables

**Table 1:** Characterization of 2-Phenylethylamine, its derivatives and opioids.

Chemical Compound	Chemical Structure	Mode of Action	Medical Properties, Adverse Effects
2-Phenylethylamine (PEA, β-phenylethylamine)	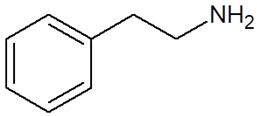	Inhibitor of monoamine oxidase B Neuromodulator Trace amine-related receptor 1 agonist Alteration of the function of the monoamine transporter (inhibiting serotonin, dopamine, epinephrine, and norepinephrine)	Accumulation in synaptic vesicles
Methamphetamine (METH)	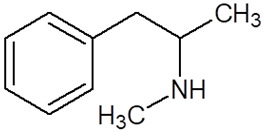	Inducement of increase in concentration of cAMP (resulting in activating the adenylyl cyclase signaling cascade and in effect increasing dopamine and glutamate in synaptic gaps)	Improvement in mood Addiction inducement Cardiovascular dysfunction (vasopressor, antihypotensive, the development of atherosclerotic plaques, and alterations in heart rhythm)
Methylphenidate	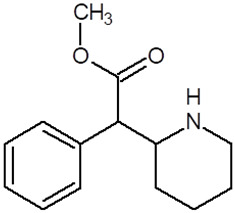	Stimulant Modulation of the activity of dopamine and norepinephrine in striatocortical regions Allosteric inhibitor of dopamine and norepinephrine reuptake	ADHD medicine Treatment in therapy of Alzheimer’s disease Improvement in mood Addiction inducement
Amphetamine	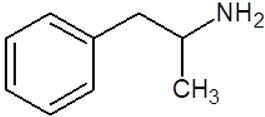	Stimulant Modulation of the activity of dopamine and norepinephrine in striatocortical regions Compensatory inhibitor of dopamine and norepinephrine reuptake	ADHD medicine Improvement in mood Addiction inducement
Methylenedioxyamphetamine (3,4-methylenedioxymethamphetamine, MDMA, ecstasy)	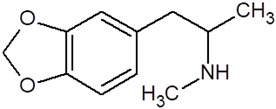	Psychedelic Hallucinogenic Inhibition of serotonin and dopamine transporters	PTSD medicine Addiction inducement Hepatic, cerebral, cardiovascular toxicity Serotonin syndrome
2,5-dimethoxy-4-methylamphetamine (DOM)	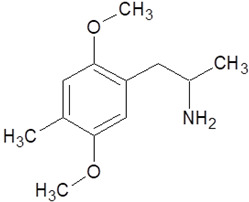	Psychedelic Hallucinogenic Inhibition of serotonin and dopamine transporters	
Mescaline	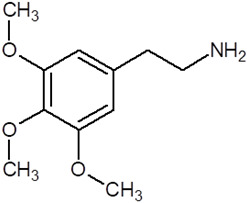	Psychedelic Serotonin 5HT_2A_ receptor agonist	Mydriasis, hyperthermia, changes in blood pressure and heart rate Synesthesia
Methadone	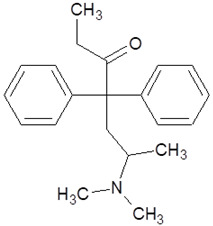	Opioid	Painkiller (in cancer and renal dysfunction treatment) Treatment of opioid dependence Respiratory depression and arrhythmias Prolongation of the QT interval
Tramadol	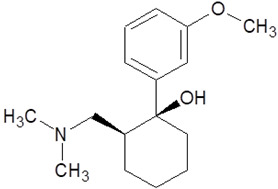	Opioid Effect on the opioid, noradrenergic, and serotonergic neurotransmitter systems (−)-tramadol: norepinephrine transport inhibitor (+)-tramadol: serotonin transport inhibitor	Painkiller Depressive and anxiety disorders medicine Reduced risk of respiratory depression and addiction Serotonin syndrome Seizures inducement
Fentanyl	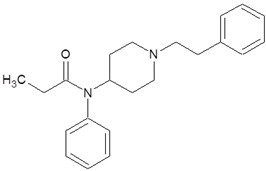	Opioid	Intraoperative anesthesia agent Painkiller Addiction inducement

**Table 2:** Findings of the studies included in the paper.

References: Author, Year	Patients	Intervention	Comparison	Outcome	Limitations	Risk of Bias
Hayley, 2023 [51]	n = 20 Healthy individuals reporting recreational stimulant use	0.42 mg/kg methamphetamine, with or without alcohol (0.38 g/kg)	RCT with placebo	Deterioration in methamphetamine group and alcohol-methamphetamine group compared to placebo, with approximately 19 times higher risk of collision	Not stated	L
Brookhuis, 2004 [55]	n = 20 Healthy MDMA users	MDMA (avg. 59 mg) with or without other substances (marijuana, alcohol, LSD)	simulation study Same population sober and after using substances	2 times higher simulated collision rate after MDMA, 2.5 times higher after polysubstance use	A quasi-experimental design	M
Madaan, 2024 [60]	n = 44 Individuals diagnosed with ADHD	Mixed-layer extended-release methylphenidate, in dose optimized individually during 21 day period	RCT Individuals treated with lisdexamphetamine dimesylate in therapeutic dose	No significant differences between lisdexamphetamine dimesylate and mixed-layer extended-release methylphenidate groups	Omission of a placebo group	L
Cox, 2006 [63]	n = 35 individuals with diagnosed ADHD	OSMOS methylphenidate, followed by extended-release mixed amphetamine salts	RCT with placebo	Participants and researchers reported noticeable driving improvements, with association in fewer past collisions	Difference in potency of MPH and amps Few participants with hyperactive subtype	L
Menefee, 2004 [69]	n = 23 Patients with chronic pain	Transdermal fentanyl, dosage 25 µg/h increasing to 50 µg/h	observational study Comparison to the same group before starting therapy	No significant deterioration in driving abilities compared to placebo group	Small sample	M
Hjälmdahl, 2012 [96]	n = 18 Healthy individuals	10 mg or 40 mg amphetamine	RCT with placebo	Improved performance after 10 mg, no enhanced or sustained effects after 40 mg	Gender inequality (only male subjects); risk of learning effect in the study	L
Kay, 2009 [97]	n = 19 Adults with diagnosed ADHD	Single-dose mixed amphetamine salts – extended release (MAS XR) 50 mg/d	simulation study Placebo, 80 mg atomoxetine	improved performance in 80% individuals on MAS XR, 40% in individuals on atomoxetine compared to placebo	Young, inexpirienced in driving population (Age: 19–25); patients with ADHD	L
Silber, 2005 [98]	n = 20 Healthy individuals	30 mg dexamphetamine	RCT with placebo	Significant deterioration compared to placebo	Not randomized tests	L
Silber, 2006 [99]	n = 20 Healthy individuals with stimulant experience	0.42 mg/kg of d-amphetamine, d,l-methamphetamine or d-methamphetamine	RCT with placebo	Low doses generally improved attention, psychomotor functions and perception speed	Not assessed baseline; brief practice sessions	M
Bosanquet, 2013 [100]	n = 30 Individuals reporting using methamphetamine at least once a week over the past three months	Participants tested for presence of psychoactive substances	observational study Individuals who reported no chronic or episodic drug use thorough their lifetime	Methamphetamine users exhibited riskier driving behavior compared to control group	Not stated	M
Stough, 2012 [101]	n = 61	MDMA in 100 mg dose or 0,42 mg/kg methamphetamine	RCT with placebo	Decrease in performance in methamphetamine and MDMA group compared to placebo	Not stated	M
Barkley, 2005 [102]	n = 52 Adults with diagnosed ADHD	One dose of 10 mg or 20 mg methylphenidate	RCT Crossover type placebo	Significant reduction in the number of impulsive errors in high dose group compared to placebo	Using a single acute dose of medication Using relatively low doses Using immediate release methylphenidate	L
Sobanski, 2008 [103]	n = 27 Individuals diagnosed with ADHD	Methylphenidate in extended release form in range: 30–60 mg	observational study Individuals with ADHD without treatment, healthy individuals without treatment	Increase in driving performance after 6-week methylphenidate treatment compared to no treatment in adults with or without ADHD	Small sample size No multiple testing	M
Cox, 2012 [104]	n = 17 Individuals with ADHD not routinely using ADHD medications	Participants randomly assigned to one of two sequences: mo treatment for 3 months followed by 3 months transdermal methylphenidate in dose 10–30 mg, or reverse order	Patients driving vehicles for 3 months without medication	During the use of transdermal methylphenidate system patients exhibited significantly fewer ADHD symptoms and experienced fewer traffic accidents compared to the period without medication	Small sample Open study	M
Verster, 2014 [105]	n = 18 Individuals diagnosed with ADHD	Individually tailored dose of methylphenidate	RCT with placebo	Methylphenidate significantly improved driving ability in adults with ADHD	Not stated	M
Aitken, 2024 [106]	n = 25 Healthy individuals	Single dose of 10 mg methylphenidate	RCT with placebo	Methylphenidate significantly improved driving ability	Low complexity of the simulated highway drive, the demands on gaze behaviour, the necessity for frequent and varied eye movements, possible ceiling effect	L
Samyn, 2002 [107]	n = 31 Healthy MDMA users	MDMA in 75 mg dose	simulation study Same population before and after administration	No significant change in performance	Not stated	L
Bosker, 2012 [108]	n = 16 Healthy individuals	Single-dose MDMA, 25 mg, 50 mg or 100 mg	RCT with placebo	No significant deterioration in driving abilities compared to placebo group	Not stated	L
Kuypers, 2006 [109]	n = 18 Healthy individuals	Single-dose MDMA, 75 mg or 100 mg, the same doses combined with alcohol	RCT with placebo	MDMA group was comparable to placebo group, MDMA + alcohol gropu performance was significantly deteriorated	Not stated	L
Dastrup, 2010 [110]	n = 16 Healthy individuals, MDMA-polisubstance users during abstinence	No MDMA administration	observational study Comparison to group never using MDMA	No significant deterioration in driving abilities in MDMA-abstinent group compared to never using group	“Nature-nurture” issues regarding patients	L
Sinclair, 2003 [111]	n = 12 Healthy individuals	General anesthesia induced by 2,5 mg/kg propofol and 1 μg/kg fentanyl or alcohol in dose inducing 0,08% BAC	simulation study The same group not recieving treatment, driving efficiency was tested in 2, 3, 4, and 24 h after inducing general anesthesia	No significant difference was observed in general anesthesia group after 2, 3, 4 and 24 h, with significant impairment in alcohol group	Results obtained without premedication and other adjuvants	L
Hayley, 2019 [112]	n = 41 Healthy individuals	0.15 mg/kg/h of ketamine for 3 h, followed by three boluses of 25 μg fentanyl	simulation study Same population before and after administration	Driving simulator results after administration of analgesic doses of ketamine and fentanyl were comparable to baseline	Not stated	M
Bramness, 2012 [118]	All Norvegians aged 18 to 69 years	Patients exposed to varying durations of methadone treatment in various form	observational study People not exposed to methadone in the same group	Exposure to methadone was associated with an approximately twice as high increase in risk of involvement in road traffic accidents resulting in bodily injury	Not stated	M
Baewert, 2007 [119]	n = 40 Opioid dependent individuals, maintained on buprenorphine or methadone for at least 2 months	Average dose of methadone 52.7 mg, and 13.4 mg buprenorphine	observational study Selected from a sample of 14,500 individuals who had previously completed 2020 standard tests for the Austrian Road Safety Board	Participants undergoing maintenence therapy performed worse in tests compared to control group	Not stated	M
Schindler, 2004 [120]	n = 30 Individuals addicted to opiates in substitutional treatment with methadone or buprenorphine	Standard doses of methadone or buprenorphine	observational study Healthy individuals without opiate-addiction history	Significantly increased errors rate and reaction time in methadone group	Not stated	M
Strand, 2019 [121]	n = 22 Healthy individuals	Two single doses of methadone (5 mg and 10 mg) and buprenorphine (0.2 mg and 0.4 mg)	RCT with placebo	Increased driving impairment in both groups compared to placebo	Not stated	L
Yang, 2021 [122]	n = 1012 Individuals with opiate use history	Undergoing methadone substitution treatment	observational study Adult opiate users not undergoing methadone substitution treatment	increase in colision risk in population undergoing methadone substitution treatment	The lack of information on road exposure Unreported or minor motor vehicle collisions not be included in the study	M
Lenné, 2003 [123]	n = 10 Patients treated with methadone	Methadone treatment with or without alcohol	observational study Individuals not using drugs	No significant differences in driving performance between methadone and control group	Not stated	L
Brubacher, 2025 [124]	n = 8328 Individuals with blood drawn within 6 h after crash	Blood obtained for research toxicology testing	observational study Drivers in whom no substances impairing the ability to drive were detected.	10.9% drivers were found driving under the influence of opiates, psychoactive substances were found in 56.% men and 52.4% women	No interview or examination of drivers Unavailability of excess blood	M
Silber, 2012 [125]	n = 20 Healthy recreational users of illicit stimulants	0.42 mg/kg of d,l-methamphetamine	RCT with placebo	No significant impairment in simulated driving performance	Not stated	L

L—Low; M—Medium; H—High.

## Data Availability

No new data were created or analyzed in this study. Data sharing is not applicable to this article.

## Supplementary Materials

The following supporting information can be downloaded at: https://www.mdpi.com/article/10.3390/ph18101555/s1, Table S1: PRISMA 2020 Main Checklist, Table S2: PRIMSA Abstract Checklist. Table S3: Studies included in qualitative synthesis; Table S4: Risk of bias assessment for individual studies, Systematic research list.

## Abbreviations

The following abbreviations are used in this manuscript:

PEA	2-phenylethylamine
ADHD	Attention-Deficit/Hyperactivity Disorder
DUID	Driving under the influence of drugs
L-AAD	L-aromatic amino acid decarboxylase
MAO-B	Monoamine oxygenase B
TAAR1	Trace amine-related receptor 1
MDMA	3,4-Methylenedioxymethamphetamine
DOM	2,5-dimethoxy-4-methylamphetamine
PTSD	Post-traumatic stress disorder
25 H-NBOMe	2-(4-iodo-2,5-dimethoxyphenyl)-N-(2-methoxybenzyl)ethanamine
25 I–NBOMe	2-(4-iodo-2,5-dimethoxyphenyl)-N-(2-methoxybenzyl)ethanamine
25 B–NBOMe	2-(4-bromo-2,5-dimethoxyphenyl)-N-(2-methoxybenzyl)ethanamine
WMC	Working memory capacity
OxyHb	Oxyhemoglobin
DeoxyHb	Deoxyhemoglobin
