# Supplementary material for: Review of Selected 2-Phenylethylamine Derivatives and Opioids, Systematic Review of Their Effects on Psychomotor Abilities and Driving Performance: Psychopharmacology in the Context of Road Safety
Source: Pharmaceuticals (Basel). 2025 Oct 16;18(10):1555. doi: 10.3390/ph18101555 (PMC12567181; doi:10.3390/ph18101555)
Supplement: Supplementary file 1 [file pharmaceuticals-18-01555-s001.zip › Supplementary systematic research.pdf]

PubMed:

(

phenethylamines[MeSH Terms]

OR amphetamines[MeSH Terms]

OR methylphenidate[MeSH Terms]

OR methamphetamine[MeSH Terms]

OR hallucinogens[MeSH Terms]

OR fentanyl[MeSH Terms]

OR methadone[MeSH Terms]

OR tramadol[MeSH Terms]

OR mescaline[MeSH Terms]

OR mdma[MeSH Terms]

OR phenylethylamine derivatives[Title/Abstract]

OR methamphetamine[Title/Abstract]

OR methylphenidate[Title/Abstract]

OR amphetamine[Title/Abstract]

OR methylenedioxyamphetamine[Title/Abstract]

OR MDMA[Title/Abstract]

OR 2,5-dimethoxy-4-methylamphetamine[Title/Abstract]

OR mescaline[Title/Abstract]

OR methadone[Title/Abstract]

OR tramadol[Title/Abstract]

OR fentanyl[Title/Abstract]

OR TAAR1s agonist[Title/Abstract]

OR psychostimulants[Title/Abstract]

OR psychoactive substances[Title/Abstract]

)

AND

(

psychomotor performance[MeSH Terms]

OR cognition[MeSH Terms]

OR executive function*[MeSH Terms]

OR attention[MeSH Terms]

OR reaction time*[MeSH Terms]

OR task performance and analysis[MeSH Terms]

OR automobile driving[MeSH Terms]

OR accidents, traffic[MeSH Terms]

OR psychomotor function*[Title/Abstract]

OR driving performance[Title/Abstract]

OR cognitive function[Title/Abstract]

OR reaction time[Title/Abstract]

OR vehicle operation[Title/Abstract]
OR driving[Title/Abstract]
OR road safety[Title/Abstract]
OR driving under influence[Title/Abstract]
OR substance use and driving[Title/Abstract]
OR drug-impaired driving[Title/Abstract]
)
AND
(
case reports[Publication Type]
OR clinical study[Publication Type]
OR clinical trial[Publication Type]
OR comparative study[Publication Type]
OR controlled clinical trial[Publication Type]
OR evaluation study[Publication Type]
OR observational study[Publication Type]
OR randomized controlled trial[Publication Type]
OR validation study[Publication Type]
)
AND
humans[MeSH Terms]
AND
English[Language]
AND
"2000/01/01"[Date - Publication] : "3000"[Date - Publication]

Embase

((
'phenethylamine'
OR 'phenethylamine derivative'
OR 'amphetamine'
OR 'amphetamine derivative'
OR 'methylphenidate'
OR 'methamphetamine'
OR 'psychedelic agent'
OR 'fentanyl'
OR 'methadone'
OR 'tramadol'
OR 'mescaline'
OR 'midomafetamine'
)/exp
OR
(

'phenethylamine derivative'
OR 'methamphetamine'
OR 'methylphenidate'
OR 'amphetamine'
OR 'midomafetamine'
OR '3,4 methylenedioxyamphetamine'
OR '2,5 dimethoxy 4 methylamphetamine'
OR 'mescaline'
OR 'methadone'
OR 'tramadol'
OR 'fentanyl'
OR 'trace amine associated receptor'
OR 'psychostimulant agent'
OR 'psychotropic agent'
):ab,ti)
AND
((
'psychomotor performance'
OR 'executive function'
OR 'reaction time'
OR 'task performance'
OR 'car driving'
OR 'traffic accident'
OR ‘attention’
)/exp/mj
OR
(
'psychomotor activity'
OR 'car driving'
OR 'reaction time'
OR 'traffic accident'
OR 'car driving'
OR 'road safety'
OR 'drunken driving'
OR 'drug driving'
OR 'driving under the influence of drug'
):ab,ti)
AND
((
'article'
OR 'clinical trial'
):it)
AND
((

'human')/exp)
AND
((
english):la)
NOT
((
'review'
OR 'systematic review'
OR 'meta analysis'
OR 'narrative review'
):ab,ti)

Web of Science:
TS=(
phenylethylamine
OR methamphetamine
OR methylphenidate
OR amphetamine*
OR methylenedioxyamphetamine
OR MDMA
OR 2,5-dimethoxy-4-methylamphetamine
OR mescaline
OR methadone
OR tramadol
OR fentanyl
OR TAAR1
OR psychostimul*
OR psychoactive substances
OR trace amine associated receptor
OR hallucinogens
)
AND
TS=(
psychomotor performance
OR cognition
OR executive function
OR psychomotor function
OR cognitive function
OR driving performance
OR driving
OR road safety
OR substance use and driving
OR drug-impaired driving
OR vehicle operation

OR reaction time
)
AND
TS=(
“case report*”
OR “clinical study”
OR “clinical trial”
OR “comparative study”
OR “controlled clinical trial”
OR “evaluation study”
OR “observational study”
OR “randomized controlled trial”
OR “validation study”
)
AND
DT=(
Article
OR Book Chapter
OR Data Paper
OR Discussion
OR Early Access
OR Proceedings Paper
)
AND
LA=(English)
AND
PY=(2000-2025)
